# PROTOCOL: Government‐Led Communication Campaigns for Reducing Violent Extremism − A Systematic Review

**DOI:** 10.1002/cl2.70031

**Published:** 2025-04-01

**Authors:** Ghayda Hassan, Sébastien Brouillette‐Alarie, Kurt Braddock, Sarah Carthy, Wynnpaul Varela, Pablo Madriaza, Paul Gill

**Affiliations:** ^1^ Department of Psychology Université du Québec à Montréal Montréal Québec Canada; ^2^ School of Criminology Université de Montréal Montréal Québec Canada; ^3^ School of Communication American University Washington District of Columbia USA; ^4^ Institute of Security and Global Affairs Leiden University The Hague The Netherlands; ^5^ Department of Psychoeducation and Social Work Université du Québec à Trois‐Rivières Montréal Québec Canada; ^6^ Department of Security and Crime Science University College London London UK

**Keywords:** government interventions, government‐led communication, mass communication strategies, systematic reviews, violent extremism prevention

## Abstract

This is the protocol for a Campbell systematic review. The main objective of this project is to gather, critically appraise, and synthesize evidence about the effectiveness of government‐led communication campaigns geared toward preventing violent extremism.

## 1 Background

Government efforts to challenge violent extremism (here, defined as adherence to beliefs, attitudes, behaviors, or intentions that support the use of violence to achieve political, ideological, social, or religious goals) are often thought to be comprised of so‐called “hardline” activities, geared toward policing, neutralization, and punishment of those who pose a threat for promoting or engaging in violence. However, as research on violent extremism has progressed, governments have come to recognize that an exclusive reliance on hardline activities is insufficient, and at times counterproductive, for reducing the threat posed by extremist ideologies and their persuasive effectiveness on vulnerable audiences. As such, governments have begun to implement other kinds of efforts to contend with the threat of violent extremism (Lum et al. 2006; Sinai et al. 2019).

As part of these alternative efforts, governments have employed a wide range of communication campaigns intended to challenge the appeal of violent extremist ideologies (see Bilazarian 2020; Braddock 2020; Ganesh and Bright 2020; Greenberg 2016; Neumann 2013; several chapters in Khader et al. 2016). Though the practices employed in the service of these efforts vary by geographic and cultural context, their common goal, broadly defined, is to diminish the persuasive appeal of ideologies that promote the use of violence to achieve socio‐political ends. In short, these communication campaigns are meant to reduce the incidence of extremist violence by challenging extremists' efforts to promote it. Well‐intentioned though these efforts may be, there exists little empirical evidence regarding their individual or collective efficacy (see Brouillette‐Alarie et al. 2022; Carthy et al. 2020; Hassan et al. 2021; Horgan and Braddock 2010; Madriaza et al. 2022; Mastroe and Szmania 2016; Pettinger 2017). A few researchers and security specialists have begun to address this issue (see Braddock 2022; Carthy and Sarma 2023; El‐Said 2015; Williams 2020; Williams et al. 2016), using experimentation and quasi‐experimentation, as well as sound monitoring and evaluation (M&E) procedures, to gauge the effectiveness of individual campaigns and practices. Still, current evidence is sparse and largely disjointed, providing little clarity in the way of the overall efficacy of government‐led, communication‐based campaigns to counter violent extremist ideologies. Moreover, there have been no attempts to determine the general effect of these efforts on target beliefs, attitudes, intentions, or behaviors. Consequently, there have thus far also been no attempts to gauge the effectiveness of the different mechanisms by which such communication efforts operate, that is, the active “ingredients or elements” that boost (or diminish) the efficacy of the campaigns or interventions.

Given that governments are increasingly turning to communication campaigns to challenge violent extremist ideologies, to effectively reduce the appeal of such ideologies, it is critical to understand the degree to which these practices are functioning as intended, if at all. This review will, therefore, synthesize existing research on the effectiveness of government‐led communication campaigns intended to challenge violent extremist ideologies and reduce radicalization to violence.

The goal of this review is to shed light on the general effectiveness of government‐led communication campaigns. More specifically, this systematic review will (a) identify government‐led communication campaigns geared toward preventing violent extremism, (b) determine the outcomes of such interventions, (c) determine which elements of communication campaigns are (or are not) effective (e.g., produce positive or negative outcomes, respectively), and (d) identify characteristics of audiences and contexts that may influence the outcomes of government‐led communication campaigns. Findings generated from the analyses in this report will help inform and support decision‐making among policymakers who wish to develop their own messaging campaigns intended to challenge violent extremist ideologies.

### 1.1 The Intervention

This systematic review aims to synthesize evaluations of government‐led campaigns with significant reach and potential impact on broader populations. As such, our analyses will revolve around campaigns evaluated at the state, provincial, or national level since these are more representative of public policies targeting systemic or large‐scale changes. In contrast, we will exclude local campaigns as these tend to focus on specific contexts or localized issues. For the purposes of this review, we furthermore define government‐led communication campaigns to counter violent extremism as any form and content of a communication‐based effort financed, developed, and implemented by a central government entity with the aim of achieving a specific objective related to the prevention of violent extremism (see definition of “communication campaign” provided by Atkin and Salmon 2010, 419; see interpretation of “government‐led” provided by Dam et al. 2023, 21). Government entities generally encompass departments, ministries, agencies, or specialized units that are directly under the control and authority of national or federal governments. In turn, campaigns typically involve the dissemination of information and aim to influence a public or target audience's opinions, perceptions, or behaviors (OECD 2021; Weiss and Tschirhart 1994). Indeed, past work on counter‐messaging campaigns shows that efforts to counter violent extremist ideologies can seek to prevent radicalization to terrorist violence or help individuals move away from terrorist groups after initial involvement (Braddock 2020). To be considered eligible for the purposes of this review, the communicative effort has to include evidence of the following:

a. Governments as the direct and overt sponsors, designers, and/or managers whereby the government and/or its subordinate organizations are explicitly mentioned. For example, in an effort to challenge the appeal of messages espoused by the so‐called Islamic State (IS) in the mid‐2010s, the U.S. Federal Bureau of Investigation (FBI) directly sponsored and launched the *Don't Be a Puppet* communication campaign and made no effort to mask its involvement (Halpern 2016; Sidahmed 2016). In 2015, the French government also launched the *Stop Djihadisme* campaign to combat the propaganda of jihadist organizations (Fragnon 2018). This campaign combined measures against the glorification of terrorism, including the closure of websites, as well as the dissemination of a “counter‐discourse” through videos and online information. Meanwhile, in Denmark, the *Aarhus Model* (implemented in the city of Aarhus) comprises several initiatives intended to undermine radicalization processes and direct individuals vulnerable to moving toward extremist violence away from doing so. In addition to the referral and counseling efforts that are part of the Aarhus Model, there is also a program involving the organization of workshops in which young Danes are warned about the dangers of violent extremism. The model also fosters open communicative dialog between Muslim communities in the city and non‐Muslims, with the goal of preventing the othering of individuals different from one another (Bertelsen 2015);
b. Objectives that aim to influence public knowledge, perceptions, and/or behaviors, such as countering misinformation and conspiracy theories, improving public knowledge of drivers to extremism, improving an audience's perceived trust in government institutions, or preventing mobilization into violence. Again, for example, the *Don't Be a Puppet* communication campaign was designed to motivate vulnerable audiences to resist the appeal of IS messaging (Halpern 2016; Sidahmed 2016);
c. A relatively large target audience (the communication must be directed toward groups and mass audiences, not specific individuals);
d. One or multiple communication channels (including nongovernmental channels). Indeed, advances in communication technologies, both in general and as specifically employed by government‐led actors, demand that we consider various media through which interventions are delivered to vulnerable audiences—in both the digital and real‐world space; and
e. One of multiple forms of communication:
  - Text‐based campaigns;
  - Audio‐based campaigns (e.g., radio, podcasts, music, presentations to groups);
  - Video‐based campaigns; and
  - Interactive media campaigns (e.g., video games).

This means that the following communicative efforts will be included:

◦ Preventing and countering violent extremism (P/CVE) media campaigns:
  ▪ Platforms:
    - Traditional media (radio, TV) campaigns (e.g., Aldrich 2014);
    - Web 1.0 campaigns (e.g., websites, MOOC);
    - Social media campaigns (Web 2.0);
    - Audio and Video applications (e.g., Spotify; TikTok), and
    - Search redirect campaigns (e.g., Redirect).
  ▪ Types:
    - **Counter‐narrative campaigns:** narratives that directly oppose and challenge the narratives proposed by violent extremist groups (Braddock and Horgan 2016);
    - **Attitudinal inoculation campaigns:** counter‐persuasive strategies whereby a communicator provides forewarning that a third party will attempt to persuade the target audience to adopt beliefs and attitudes that are different from their own (McGuire 1961);
    - **Alternative narrative campaigns:** narratives that undermine the assumptions of another narrative without directly engaging with its content (Carthy 2022). For example, the Shared Values Initiative (Kendrick and Fullerton 2004) was a campaign initially piloted by the United States government across several Muslim countries in the post‐9/11 era. The campaign creates videos which attempted to promote what the government was “for” rather than against without directly challenging specific narrative components (e.g., depictions of Muslims living happily in the US to communicate themes of openness, diversity, and inclusiveness); and
    - **Public awareness campaigns:** narratives that inform on VE risks without countering a specific discourse (e.g., COVID campaigns).

◦ Educational/school campaigns only when led by central government entities:
  ▪ Violence prevention campaigns with a P/CVE element/objective;
  ▪ Civic education with a P/CVE element/objective; and
  ▪ Digital literacy campaigns with a P/CVE element/objective.

◦ “Suspicious activities” reporting campaigns (e.g., *See Something, Say Something*).
  This means that the following will be excluded:
◦ Any one‐on‐one intervention (e.g., direct one‐on‐one messaging P/CVE campaigns; see Frenett and Dow 2015);
◦ Individual psycho‐social support (e.g., therapy; community‐based support interventions);
◦ Local community or school campaigns or interventions; and
◦ Messages tailored for individuals rather than groups.

Again, these different examples are the most well‐represented communication campaigns in the literature but should not be considered a comprehensive account of all intervention types and modalities. Should we identify other types of campaigns, we will incorporate them into our search, selection, and analyses.

Although the systematic review will cover government‐led communication campaigns, this should not imply that all campaigns included in the review will be specifically implemented or delivered by government officials or a central entity. Although this may be the case for some interventions, there exists a great number of campaigns that—though financially and logistically led by governments—are delivered by others. The implementers of these campaigns can include civil society organizations, local community organizations, and other nongovernment entities. These campaigns will be included as long as there is a clear and direct reference to the government entity in question. To be considered “government‐led,” these campaigns must explicitly mention or acknowledge the involvement, direct funding (not through a third party), guidance, or authorization from a government or one of its departments, ministries, agencies, or central bodies. That government entity must have been involved in the planning, initiation, or oversight of the communication campaign. The acknowledgment must provide evidence that the government, even if not directly implementing the campaign, was actively involved or associated with it in a significant manner.

### 1.2 How the Intervention Might Work: Developing the Logic Model

Researchers in the fields of criminology and psychology have largely dominated the discussion related to how violent extremist ideologies might be countered. Experts in criminology and political science have conducted a significant amount of research to show how policing and securitization can affect the contagion of violent extremism by removing those who espouse extremist ideologies from their positions of influence (e.g., Hamilton 2019; Jordan 2019). By contrast, experts in psychology have explored how individuals might be made resilient to violent extremist ideologies or abandon them altogether (e.g., Silva and Deflem 2020). Whereas the former work relates more closely to the aforementioned “hardline” counterterrorism activities, the latter is characterized by so‐called “soft” approaches and rooted in familiar, psychological frameworks, such as dual process models of cognition (Evans and Stanovich 2013) or resilience and resistance‐based models (Knowles and Linn 2004). It is within the latter domain that government‐led, communication‐based efforts to challenge violent extremist ideologies generally operate.

Aside from criminology and psychology, there is some scholarship within media studies and communication that might provide some insight into how audiences engage with salient messages. Although consideration of all theories from media studies is beyond the scope of the current review, there are three theoretical perspectives—framing theory, agenda‐setting theory, and cultivation theory—that can inform our understanding of how messages might be interpreted in the context of undermining violent extremism. Adherents to framing theory (originally in Goffman 1974; Snow et al. 1986) have long contended that strategic communicators can elect to emphasize or de‐emphasize certain elements of a story to affect audience perceptions of it. For instance, P/CVE messaging may highlight violence performed by a militant group and fail to mention that the group also provides protection to vulnerable citizens. This kind of message would “frame” the militant group as exclusively malicious.

Agenda‐setting theory (originally McCombs and Shaw 1972) similarly contends that distributors of messages can influence audience perceptions of how important an issue is by electing to inform those audiences (or not) about those issues. Using the same example as above, counter‐extremism campaigns may comprise a number of different media messages, all of which discuss a militant group's actions as religious impropriety. These messages would collectively “set the agenda” that religion is an important theme to consider when evaluating the validity of the group.

Finally, cultivation theory (originally Gerbner 1973) argues that audience perceptions about the world are affected by how often they encounter certain themes in the media. For instance, a campaign may produce a large number of messages that illustrate the violent actions performed by the militant group. In doing so, audiences to these messages would perceive the group to be a threat to their own safety.

Despite a growing corpus of research that informs on how radicalization to violence or extremist messaging influences radicalization into violent extremism (Hassan et al. 2018; Madriaza et al. 2024), there is a general dearth of understanding about how different kinds of communication‐based interventions might be received by their intended audiences. Also, while there are reports and guidelines offering recommendations on how to develop P/CVE campaigns (e.g., Carthy 2022), there has been limited investigation into the positive or unintended negative outcomes of such campaigns in the realm of violent extremism (e.g., Byrne and Hart 2009; Zhao and Fink 2021).

**Figure 1:**
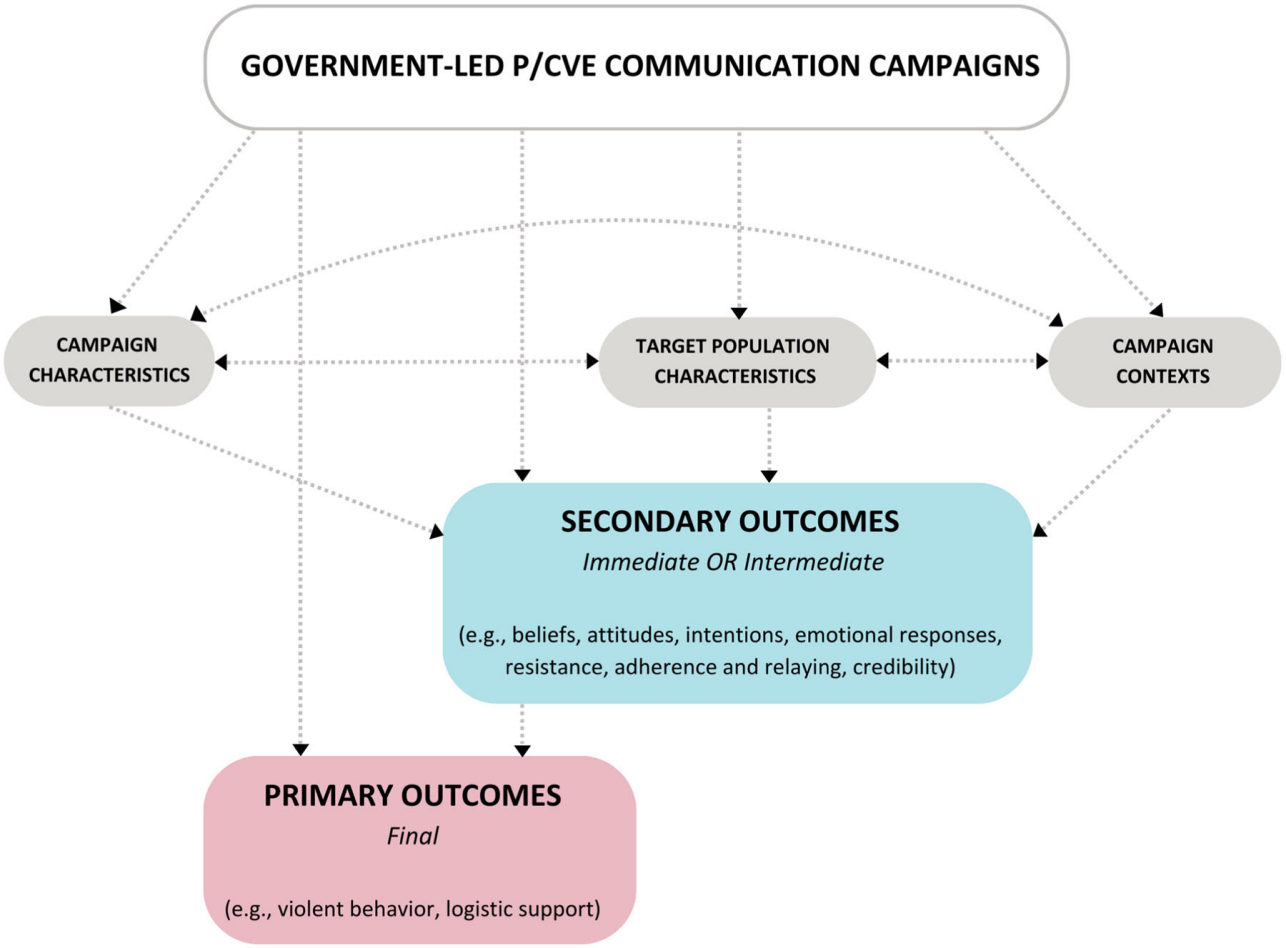
Logic model for government‐led campaigns: What works, for whom, in which context, and how.

That being said, and to better understand what the effects of the exposure to a government‐led P/CVE campaign are and how they might work, we developed a logic model that will provide a clearer basis to illustrate these relations, as well as refine them as the data coding and analysis progresses (see Figure 1).

Our logic model takes into account the fact that different campaigns may have different objectives, be tailored to different audiences, have different contents, take different forms, and use different channels. Government‐led campaigns will be associated with a diversity of psychological (i.e., cognitive, emotional) and behavioral responses (outcomes) that can be positive (benefits) or negative (unintended harms or iatrogenic effects). In all cases, such outcomes will likely be the result of interactions between campaign characteristics, target audience characteristics, and the characteristics of the wider social context where these campaigns occur (e.g., political in/stability, violent attacks, or absence of). Secondary outcomes encompass both immediate and intermediate outcomes. The former may involve target audience uptake of the campaign, emotional responses to it, or increased or unintended publicity of extremist groups or narratives. The latter can include a change in extremist attitudes or intentions and increased (or decreased) trust in government. Primary outcomes, meanwhile, refer to the final outcomes of a campaign, such as a measurable decrease in violent behaviors related to extremism.

While there is a dearth of M&E efforts in the P/CVE field on government‐led campaigns, literature on such campaigns in other areas of risk behaviors lends support to our logic model, as well as provides some evidence of the potential effectiveness of such campaigns. For example, systematic reviews on alcohol‐impaired driving (Yadav and Kobayashi 2015), illicit drug use (Allara et al. 2015), or smoking‐related campaigns (Lim et al. 2024; Perman‐Howe et al. 2022; Sadeghi et al. 2020), all show some positive changes in knowledge, attitude, and behaviors, but also potential iatrogenic effects. In addition, their efficacy depends on how they are implemented, the populations they target, and whether they are coupled with other complementary actions (Allara et al. 2015; Lim et al. 2024; Perman‐Howe et al. 2022; Sadeghi et al. 2020).

Finally, the likely mechanisms of effectiveness of government‐led campaigns in the P/CVE space will occur via psychological processes. For instance, campaigns that hinge on narrative‐based messaging are likely to promote psychological transportation (the tendency for audiences to feel “caught up” in a story, leaving them less likely to engage in counter‐arguing; Green and Brock 2002), identification with characters in the narratives (the tendency for audiences to perceive themselves as similar to characters, thereby becoming sympathetic to their views; De Graaf et al. 2012), parasocial interaction (the tendency for audiences to perceive themselves as in a relationship with characters, thereby becoming sympathetic to their views; Giles 2002), and/or emotional processing (Appel and Richter 2010). Though the study of inoculation in the realm of violent extremism has only begun recently, the data show it to be an effective means of preventing persuasion by extremist propaganda. Specifically, inoculating against violent extremist propaganda has been shown to (a) increase psychological reactance (Brehm and Brehm 1981) in response to the propaganda, (b) reduce audience perceptions of the extremist group's credibility, (c) reduce audience gratification in response to the propaganda, and (d) reduce audience intentions to support the group that produces the propaganda (see Braddock 2022; Carthy and Sarma 2023). While we do not have the objective of systematically reviewing these mechanisms, we will summarize them narratively when mentioned or assessed by study authors.

### 1.3 Why It Is Important to Do the Review

The proliferation of sophisticated communication strategies employed by violent extremists invites scrutiny regarding the effectiveness of government‐led communication strategies designed to counter them. Although governments have grown more adept at developing efforts to challenge violent extremist ideologies, government‐based interventions—communication‐based or otherwise—have historically gone empirically unexamined (see Williams 2020). Moreover, efforts to evaluate the efficacy of government‐led, communication‐based efforts to counter violent extremist ideologies have been hampered by methodological difficulties (e.g., establishing causality), bureaucratic hurdles (e.g., pressures to report questionable data or, conversely, *not* to report data that would look unflattering for government agencies), and a lack of consistency across campaigns (Holmer et al. 2018). To date, empirical investigations have been largely limited to two approaches. They have either listed counter/alternative narrative campaigns and review reports or conducted single‐study evaluations of counter‐radicalization campaigns with a communication component employed in specific social conditions (e.g., Brouillette‐Alarie et al. 2022; Cherney and Belton 2021; Harris‐Hogan 2020; Hassan et al. 2021; Helmus and Klein 2019; Mastroe and Szmania 2016). For example, whereas Carthy et al. (2020) explored the efficacy of counter‐narratives for preventing radicalization to violence, Braddock (2022) has discussed the effectiveness of attitudinal inoculation as a means of challenging violent extremist ideologies. Recently, there has been a rise in the number and quality of evaluation studies, some of which (e.g., Bilali 2022) have robust methodologies such as Randomized Control Trials (RCTs).

The ability to synthesize the literature on government‐led communications using robust methodologies is highly relevant for many reasons. First, this will prevent the proliferation of empirically unfounded recommendations and faulty implementations of communication practices, with the risk of resulting in counter‐productive results. Second, governments are accountable to their populations, and in addition to legal and ethical considerations, they have the responsibility to prevent unintended harms from their initiatives, particularly in a domain that involves public safety and national security. Knowing what works in this field will also help reduce the waste of financial resources and improve the allocation of public funds, improve government transparency and associated trust in its institutions, and ultimately help develop more efficient campaigns to reduce the threat of violent extremism.

This review will help us, in short, to understand “what works, for whom, in which context, and how?” (Pawson 2002, in Gielen 2019, 1150).

### 1.4 How This Review Might Supplement What Is Already Known in This Area

To properly contextualize our work, we searched for prior relevant systematic reviews and meta‐analyses in ERIC, Academic Search Complete, and Google Scholar, using keywords and concepts relevant to our study. Similarly, we searched for existing published reviews on the subject in the Campbell Library, the Cochrane Library, and the PROSPERO registry.

We found one systematic review conducted by Carthy et al. (2020) on the efficacy of counter‐narratives for the prevention of radicalization to violence. The review included 19 studies. The studies reported on counter‐narrative campaigns that aimed to undermine the narrative(s) of hostile social constructions of an out‐group through the use of “stereotype‐challenging, prosocial, or moral ‘exemplars’, or alternative accounts, inoculation and persuasion” (p. 1). The results of the review showed that overall, the counter‐narrative interventions had a small effect on reducing risk factors for radicalization to violence. The authors further reported that some approaches (e.g., counter‐stereotypical exemplars) were effective “at targeting realistic threat perceptions, in‐group favoritism and out‐group hostility” (p. 2). However, other theoretical approaches, such as persuasion, prompted resistant responses. More broadly, there was no clear reduction in symbolic threat perceptions, implicit bias, or intent to act violently.

In systematic reviews on the efficacy of primary, secondary, and tertiary prevention interventions in the field of P/CVE, Hassan et al. (2021) and Brouillette‐Alarie et al. (2022) found studies looking at online intervention interventions for individuals on a violent radical trajectory or those that were members of online extremist groups. The evaluation studies of counter‐narrative campaigns in which individuals were directly contacted via messaging apps or sites often led to defensive responses and security concerns for practitioners, which made the positive outcomes pale in comparison to the iatrogenic effects associated with these campaigns. However, counter‐narrative campaigns displaying ads in front of Google searches for violent radical materials seemed to result in less defensive responses from concerned individuals.

This review will thus address the gap in the empirical literature for P/CVE in two key ways. First, primary meta‐analyses will synthesize existing quantitative evidence on the efficacy of communicative efforts, thereby demonstrating what works to diminish the appeal of violent extremist ideologies. Second, and just as importantly, moderator analyses of extant practices will illustrate the conditions—cultural, geographical, political, audience‐related, content‐related, and otherwise—under which specific practices are most effective. Thirdly, even though qualitative studies will not be included in the summary of results, the review of qualitative evaluation studies will provide important contextual information to help interpret the results of quantitative studies and inform conclusions and recommendations. Collectively, the results of this review will inform future decision‐making about the design, implementation, and evaluation of government‐led communication campaigns by summarizing the evidence for their efficacy and providing insight that will allow stakeholders to optimize the allocation of limited resources and improve the efficiency of future communication‐based efforts to prevent violent extremism.

## 2 Objectives

The primary objective of this review is to summarize available evidence on the effectiveness of government‐led communication campaigns that are intended to challenge, undermine, counteract, or neutralize violent extremist ideologies among diverse audiences. In doing so, this review seeks to determine whether and the degree to which government‐led communication campaigns effectively prevent audiences from developing beliefs, perceptions, attitudes, intentions, emotions, or behaviors consistent with violent extremist ideologies, or in improving audiences' beliefs, perceptions, attitudes, intentions, or behaviors consistent with positive social interactions that weaken the appeal of said ideologies. In addition, by exploring the details associated with government‐led communication campaigns, this review will also identify whether and the degree to which intervention‐specific (e.g., form, modality) and/or audience‐specific (e.g., cultural affiliation) factors moderate the effectiveness of the interventions.

More specifically, this systematic review will do the following:

a. Identify government‐led communication interventions geared toward preventing or countering radicalization to violence/violent extremism;
b. Determine salient outcomes of such interventions;
c. Determine which elements of communication interventions are (or are not) effective (e.g., produce positive, negative, or negligible outcomes); and
d. Identify characteristics of audiences and contexts that may influence the outcomes of government‐run communication interventions.

Given that the systematic review will provide evidence for or against the effectiveness of some communication practices, a secondary objective of the review will be to provide insight into which of those practices are effective to policymakers. As such, this review aims to support policymakers in determining the most effective ways to design communication‐based campaigns tailored to specific implementation contexts. It will also help policymakers shape policies related to funding and programming, help establish more focused priorities for prevention campaigns targeting diverse audiences, and facilitate the evaluation of existing PVE policies.

In addition to identifying specific campaigns that are eligible for inclusion in our analyses, it is also important to note the ethical issues concerning the discriminatory practices inherent in how some of these campaigns are delivered (Bjorgo and Gjelsvik 2015; Eijkman 2011; Williams et al. 2016). As such, when possible, we will note both methodological and ethical issues associated with any campaigns identified as eligible for inclusion in the analyses.

## 3 Methods

### 3.1 Criteria for Considering Studies for This Review

#### 3.1.1 Types of Studies

To fulfill the objectives of this review, included studies must meet several inclusion criteria. Specifically, included studies must explore interventions that are developed and/or administered by a government or government‐supported agent and feature the use of some control against which the efficacy of the intervention is measured (this includes pre‐/post‐measures). Details related to these, and other inclusion criteria, are discussed in Sections 3.1.2–3.1.6 below.

Given the review's focus on the effectiveness of government‐led, communication‐based campaigns, we will incorporate all available studies that utilize experimental designs and quasi‐experimental comparison group designs that provide an estimate of the effectiveness of the intervention. Stated differently, we will include all studies from which we can draw conclusions about the impact of an intervention on audience‐specific outcomes.

We will include all studies that feature RCTs. Although RCTs (i.e., pure experimentation) are the gold standard for determining causality, rigorous quasi‐experimentation can also be used to determine causality (Farrington 2003; Shadish et al. 2001). In this vein, and consistent with a previous Campbell systematic review conducted by Carthy et al. (2020), we will also incorporate the following “strong” quasi‐experimental and other study types:

- Factorial designs, with more than one independent variable (e.g., pre‐post as a within‐subjects variable, and exposure (e.g., present/absent) as a between‐subjects variable; and
- Single‐group pre‐ and post‐test studies that collect data at baseline and after the exposure to the campaign.

#### 3.1.2 Types of Participants

Given that government‐led communication campaigns are tailored to audiences of all types, to gauge the effectiveness of such interventions, this review will include studies that make use of samples from populations of any age, gender, ethnicity, cultural identity, religion, national citizenship, or other demographic characteristics. However, it will exclude targeted communications. Moderator analyses will be performed to see if participants' characteristics (e.g., age, gender, religion, ethnicity) and contexts (e.g., region, sector, etc.) have an effect on the intervention's outcomes.

#### 3.1.3 Types of Interventions

To qualify for inclusion in this review, studies must explore the results of a government‐led, communication‐based intervention to challenge a violent extremist ideology. The ideology being countered can have any focus (e.g., jihadi, far‐right), but must incorporate at least one element that advocates violence as a viable means of achieving political change. Section 1.2 (The intervention) covers in detail the types of communication‐based interventions that are eligible for the purposes of this review.

#### 3.1.4 Types of Outcome Measures

Because violent extremist ideologies are meant to prompt specific kinds of thinking and actions, studies that investigate the effect of government‐led communication campaigns on the persuasiveness of violent extremist ideologies will be included. As such, included studies will evaluate the effects of such campaigns on both primary and secondary outcomes. Even though we anticipate that secondary outcomes will be more numerous and, thus, harder to collapse together, efforts will be deployed to aggregate them in a clear and consistent way. See Table 1 for a synopsis of some categories of outcomes. Definitions for these phenomena are offered in Sections 3.1.4.1 and 3.1.4.2.

**Table 1:** Salient dependent variables.

Primary outcomes	Secondary outcomes
Diminution of **violent behavior** supporting a violent extremist group or ideology (e.g., “Have you ever decided against traveling overseas to provide support to [terrorist organization]?”)	Beliefs inconsistent with violent extremist ideology (e.g., rejection of false statements described in extremist propaganda).
Diminution of **logistic support** for a violent extremist group (e.g., “Have you ever refrained from donating money to a cause or charity in support of [terrorist organization]?”)	Attitudes inconsistent with violent extremist ideology (e.g., trust toward an out‐group or government)
	Intentions inconsistent with violent extremist ideology (e.g., commitment to nonviolence)
	Salient emotional responses (e.g., reduced anger or frustration)
	Resistance to violent extremist propaganda
	Questioning the credibility of violent extremist groups

##### 3.1.4.1 Primary Outcomes

Because the end goal of communication campaigns is to reduce support for or engagement in violent activity, our primary outcomes of interest will relate to manifest violence. These include manifest violent behavior and logistic support for a violent extremist group (or in support of a violent extremist ideology):

- **Violent behavior:** manifest violence in support of a violent extremist group or ideology.
- **Logistic support:** manifest support that facilitates an extremist group's ongoing operations.

##### 3.1.4.2 Secondary Outcomes

Although diminution of violent behavior and support for such behavior is the “gold standard” of prevention efforts, there are some outcomes that research has shown to precede primary outcomes. These outcomes are the development of beliefs, attitudes, and intentions consistent with an ideology that supports violent activity. Definitions for these terms are as follows:

- **Beliefs:** unvalenced (i.e., no value attached) perceptions of the world (see Fishbein and Ajzen 1975).
- **Attitudes:** valenced (i.e., value attached) judgments of the world (see Fishbein and Ajzen 1975).
- **Intentions:** stated readiness and/or willingness to support a violent extremist group logistically or with violence (see Fishbein and Ajzen 1975).

In addition to these, we wish to measure other outcomes that may relate to an individual's predilection for or aversion to engaging in violence following exposure to government‐led communication‐based campaigns. These outcomes largely relate to the development of resistance to extremist messaging and include emotional response, resistance to messaging, and credibility attribution:

- **Emotional response:** the degree to which an individual experiences various kinds of affect in response to extremist messaging (e.g., fear, pride, happiness, sadness).
- **Resistance to propaganda:** anger and counter‐arguing (combined as reactance) in response to extremist propaganda (see Brehm 1966).
- **Credibility attribution:** the degree to which an individual finds the source of an extremist message to be trustworthy.

#### 3.1.5 Duration of Follow‐Up

Because government‐led communication campaigns often differ in duration—both in terms of their implementation and follow‐up with participants—the systematic review will not exclude any studies based on time between exposure to the government‐led campaigns and observation of outcomes.

#### 3.1.6 Types of Settings

Studies will not be excluded based on the setting in which the intervention was implemented; both lab and field studies will be considered eligible for inclusion. They will be meta‐analyzed separately, however.

#### 3.1.7 Inclusion and Exclusion Criteria

Considering the above and based on our logic model, this systematic review will use the following inclusion and exclusion criteria:

**Inclusion criteria:**

1. Must be an eligible type of communication campaign.
  a. Interventions must target the entire population or large groups of audiences (several hundreds to millions) with messaging being tailored to the audience, for example, information campaigns on social media, messaging campaigns on government websites, ads on radio and television, messaging campaigns in education and work settings, and so forth (see Section 1.2).

2. The campaign/intervention must aim to lower adherence to, among other things, violent extremist outcomes.
  a. For eligible outcomes, see Table 1.

3. The campaign/intervention must be government‐led, for example:
  a. The campaign is created in collaboration in terms of content and modalities with a central government entity;
  b. The campaign is distributed by a central government entity;
  c. The campaign has the logo of the government or one of its central entities;
  d. The authors or publishers consider the campaign to be government‐led; or
  e. Other criteria noted during the data extraction phase that indicate the campaign/intervention is government‐led.

4. The study is empirical and quantitative with designs that comprise data as follows:
  a. Publications adopt an experimental or quasi‐experimental design where at least one of the independent variables involves comparing the recipients of a communication campaign to a control or comparison exposure. These may include:
    i. RCTs;
    ii. Factorial designs; or
    iii. Single‐group pre‐ and posttest studies which collect data at baseline and after exposure to the campaign
  b. The quantitative section of mixed‐methods studies will be eligible, provided that they meet criterion 4a; and
  c. The study must comprise primary data.

**Exclusion criteria:**

1. Exclude P/CVE interventions:
  a. that are not communication campaigns (e.g., one‐to‐one psychological interventions, rehabilitation programs, etc.);
  b. that do not aim, among other things, to lower adherence to violent extremist outcomes (e.g., a government ad campaign to prevent drunk driving).
  c. that do not have governments as the direct and overt sponsors, designers, and/or managers of the communicative efforts; or
  d. whose only link to government entities is funding.

2. Exclude designs that are not experimental, quasi‐experimental, or use factorial designs (e.g., qualitative designs or expert opinion; secondary data studies, such as meta‐analyses and systematic reviews).

### 3.2 Search Methods for Identification of Studies

Studies will be identified by searching salient databases (see Section 3.2.1) with search term blocks consistent with our exclusion and inclusion criteria. Specifically, four themes (search term blocks) will need to be present (united by AND) for a record to be considered (see Table 2). The search will be done in the title, abstract, keywords, and subject/indexing fields and will cover research published until December 31st, 2024. Searches will be conducted in English, but no languages will be excluded from the results (i.e., if a Spanish paper contains an English abstract that is identified by our English search string, it will be included). Endnote will be used to index the results. Sample search results from four platforms—EBSCO, ProQuest, Web of Science, and PsycINFO—are provided in Appendices S1A–S1D.

**Table 2:** Search syntax.

Themes/search term blocks	Search syntax
Communication‐based intervention	(
	((messag* OR communic* OR fram* OR narrative* OR argument*)
	W/3
	(initiative* OR program* OR project* OR campaign* OR interv* OR counter* OR contest* OR alter* OR anti OR inoculat* OR persua* OR dissua* OR awareness OR inform* OR educat* OR prevent*))
	OR
	((initiative* OR program* OR project* OR campaign* OR interv*)
	W/3
	(counter* OR contest* OR alter* OR anti OR inoculat* OR persua* OR dissua* OR awareness OR inform* OR educat* OR prevent*))
	OR
	(campaign*)
	)
AND	
Government‐led	(council* OR government* OR minist* OR state* OR provinc* OR nation* OR department* OR federal*)
AND	
Radicalization to violence/extremism, PVE, CVE	(radicali* OR “alt left” OR “alt‐left” OR “alt right” OR “alt‐right” OR anarch* OR anticapitalis* OR “anti‐capitalis*” OR “anti capitalis*” OR antifas* OR “anti fas*” OR “anti‐fas*” OR “anti islam*” OR “anti‐islam*” OR “anti muslim*” OR “anti‐muslim*” OR antisemiti* OR “anti‐semiti*” OR “anti semiti*” OR blackpill OR “black‐pill” OR “black pill” OR ecoterror* OR “eco‐terror*” OR “eco terror*” OR ecoviolen* OR “eco‐violen*” OR “eco violen*” OR “environmental* violen*” OR “extreme right” OR extremis* OR fanatici* OR “far left” OR “far‐left” OR “far right” OR “far‐right” OR fascis* OR “foreign fight*” OR fundamentalis* OR “hate crime*” OR “ideological* violen*” OR incel OR indoctrinat* OR insurgen* OR islamis* OR islamophob* OR jihadis* OR “left wing” OR “left‐wing” OR “male supremac*” OR “mass shoot*” OR misogyn* OR neonazi* OR “neo nazi*” OR redpill OR “red‐pill” OR “red pill” OR “right wing” OR “right‐wing” OR salafi* OR “school shoot*” OR “scientific racis*” OR supremis* OR terroris* OR “white supremac*” OR zionis*)
	OR (lone W/2 (actor* OR offend* OR wolf*))
	OR (violen* W/2 (political* OR racis* OR religio* OR separatis*))
	OR (radical* W/2 (group* OR ideolog* OR left* OR movement* OR right*))
	OR (suicid* W/2 (attack* OR bomb*))
	OR (violen* W/2 (racis* OR separatis*))
AND	
Evaluation of the intervention and/or its components	(evaluat* OR assess* OR apprais* OR effectiv* OR efficac* OR investigat* OR impact* OR experiment* OR trial* OR quasiexperiment* OR “quasi‐experiment*” OR “quasi experiment*” OR random* OR RCT OR analy* OR measur* OR outcome*)

#### 3.2.1 Electronic Searches

We will conduct searches in a variety of bibliographic databases, both subject‐specific databases and general multidisciplinary databases. While the searches will employ standard Boolean logic, they will be tailored to the features of each database, making use of available controlled vocabulary. All academic databases to be searched are included in Table 3, with gray databases in Table 4.

**Table 3:** Academic databases.

Host platform	Database
EBSCOHost	Academic Search Complete
	Communication and Mass Media Complete
	Communication Abstracts
	Criminal Justice Abstracts
	Military and Government Collection
	PsycARTICLES
	PsycEXTRA
	Psychology and Behavioral Sciences Collection
	PsycINFO
ProQuest	Applied Social Sciences Index and Abstracts (ASSIA)
	Criminal Justice Abstracts
	Dissertations and Theses Index
	International Bibliography of the Social Sciences (IBSS)
	National Criminal Justice Reference Service (NCJRS)
	Policy File Index
	ProQuest Criminal Justice
	ProQuest Political Science
	ProQuest Social Science
	ProQuest Sociology
	Social Services Abstracts
	Sociological Abstracts
	Worldwide Political Science Abstracts
Web of Science	Conference Proceedings Index: Social Sciences and Humanities
	Emerging Sources Citation Index
	Social Sciences Citation Index
Informit	CINCH: Australian Criminology Database
Wiley	Campbell Systematic Reviews
Other	Global Policing Database (https://gpd.uq.edu.au/)
	Cochrane Database of Systematic Reviews
	European Commission
	Ingenta Connect
	JSTOR
	Journals@Ovid
	Oxford Journals Online
	Scopus

**Table 4:** Gray databases.

URL	Database
https://cup.columbia.edu/reference/ciao	Columbia International Affairs Online (CIAO)
https://www.coe.int/en/web/portal	Council of Europe
https://njlaw.rutgers.edu/cj/gray/search.php	Don M. Gottfredson Library of Criminal Justice Gray Literature
https://www.govinfo.gov/#	GovInfo
https://www.hsdl.org/c/	Homeland Security Digital Library (HSDL)
https://llmc.com/	LLMC Digital
https://muse.jhu.edu/	Project Muse
https://www.ssrn.com/index.cfm/en/	Social Science Research Network
https://trackingterrorism.org/	Terrorism Research and Analysis Consortium (TRAC)

#### 3.2.2 Searching Other Resources

We will also explore other electronic avenues for identifying salient research. These efforts will include the following:

- Searching counterterrorism organization websites and databases (see examples of websites in Table 5);
- Searching trial registries (see Table 6);
- Backward and forward citation searching on all included studies; and
- Reviewing academic journals from 2000—present in journals that are non‐indexed or were not indexed at any point during this time period. We have chosen to focus on articles published after 2000 as our preliminary searches show minimal relevant material before this period. Also, violent extremism has evolved significantly, and campaigns before 2000 do not address the same phenomenon.

**Table 5:** Counterterrorism organization websites and databases.

Organization	Website	Country of origin
Center on Terrorism, Extremism, and Counterterrorism	https://www.middlebury.edu/institute/academics/centers-initiatives/ctec	United States of America
Combating Terrorism Center (West Point)	https://ctc.usma.edu/	United States of America
Countering Violent Extremism Evaluation Tool	https://www.cveevaluation.nsw.gov.au/home	Australia
Department of Homeland Security	https://www.dhs.gov	United States of America
European Union	https://www.coe.int/en/web/portal/home	Multinational
Global Center on Cooperative Security	https://www.globalcenter.org	Multinational (United States, United Kingdom, Kenya, Belgium)
Global Terrorism Research Center (Monash University)	https://www.monash.edu/arts/social-sciences/gtrec	Australia
Hedayah	https://www.hedayahcenter.org	United Arab Emirates
Impact Europe	https://impacteurope.eu	European Union
Institute for Strategic Dialog (ISD Global)	https://www.isdglobal.org/extremism/	Multinational (based in the United Arab Emirates)
National Consortium for the Study of Terrorism and Responses to Terrorism (START)	https://www.start.umd.edu/	United States of America
National Counterterrorism, Innovation, Technology and Education Center (NCITE)	https://www.unomaha.edu/ncite/index.php	United States of America
Norwegian Defense Research Establishment	https://www.ffi.no/en	Norway
Prev‐Impact Canada	https://prev-impact.ca/	Canada
Public Safety Canada	https://www.publicsafety.gc.ca/index-en.aspx	Canada
Radicalization Awareness Network (RAN)	https://ec.europa.eu/home-affairs/what-we-do/networks/radicalisation_awareness_network_en	European Union
RAND (and RAND Europe)	https://www.rand.org	Multinational
Royal United Services Institute (RUSI)	https://rusi.org/publications	United Kingdom
Search for Common Ground	https://www.sfcg.org/	Multinational
Terrorism Research Center (University of Arkansas)	https://terrorismresearch.uark.edu/	United States of America
The Global Counterterrorism Forum	https://www.thegctf.org	Multinational
Triangle Center on Terrorism and Homeland Security	https://sites.duke.edu/tcths/	United States of America
U.S. Agency for International Development (USAID)	https://www.usaid.gov/	United States of America
UN‐CTED	https://www.un.org/securitycouncil/ctc/	Multinational
UNESCO	https://www.unesco.org/en	Multinational
United Nations Development Program (UNDP)	https://www.undp.org/	Multinational
VOX‐Pol	https://www.voxpol.edu	European Union

**Table 6:** Trial registries.

Organization	Website	Country of origin
Cochrane Central Register of Controlled Trials (CENTRAL)	https://www.cochranelibrary.com/central/about-central	United Kingdom
ISRCTN Registry	https://www.isrctn.com/	United Kingdom
Trials Register of Promoting Health Interventions (TRoPHI)	https://eppi.ioe.ac.uk/webdatabases4/Intro.aspx?ID=12	United Kingdom
UK Clinical Research Network (UKCRN Study Portfolio)	https://www.ukcrc.org/research-infrastructure/clinical-research-networks/uk-clinical-research-network-ukcrn/	United Kingdom
Unreported Trials Register		N/A

In addition to these databases and other electronic sources, we will also try to collaborate with major international and national government agencies in the field of countering extremism, when possible and contacts permitting, to gain access to unpublished or gray literature evaluation reports (e.g., ministries of public safety or national security; UNOCT; UN‐CTED).

Additional strategies for identifying salient resources will include harvesting the reference lists of Campbell Reviews, Cochrane Reviews, and other relevant reviews on similar topics.

### 3.3 Data Collection and Analysis

#### 3.3.1 Description of Methods Used in Primary Research

The relative scarcity of empirical data on the effectiveness of government‐led, communication‐based government interventions makes it difficult to predict the kinds of studies that will form the majority of those collected. Still, the nature of the interventions provides some guidance on the kinds of methods that have been used to judge their efficacy in the past. For studies conducted by governments themselves, we anticipate that most data will be presented in the form of recidivism rates and retrospective analyses of intervention participants' beliefs and attitudes. For studies conducted by independent researchers, however, it is plausible that non‐intervention comparison groups can be retroactively constructed to gauge the effectiveness of the intervention on specific outcomes. In this way, we anticipate that quantitative studies of government‐led, communication‐based interventions will be quasi‐experimental in kind. We expect true experiments (i.e., experiments that use a pure control group from the intervention outset) to be rare.

#### 3.3.2 Criteria for Determination of Independent Findings

Our unit of analysis will be an effect size representing a specific association or causal relationship between exposure to a government‐led communication campaign and a potential outcome. However, because government‐led, communication‐based interventions typically target several outcomes, we expect that some studies that report on these interventions will describe effect sizes in relation to multiple dependent variables. To ensure that all effect sizes included in this review are derived from statistically independent findings and do not disproportionately influence the final calculated effect size—a key challenge in meta‐analysis (Lipsey and Wilson 2001)—we will take measures to minimize or eliminate potential dependencies among effect sizes.

First, we will catalog the outcomes reported in each study—along with their operational definitions—and organize them into categories suitable for synthesis in a meta‐analysis (cf. Table 1 for salient dependent variables). Second, we will review sample information so that identical outcomes relating to the same sample are not included in the same meta‐analysis. For studies reporting duplicate outcomes from the same data set, we will prioritize the peer‐reviewed publication that offers the most detailed information. If, however, the study reports on outcomes that are conceptually similar, we will use the robust standard errors method of Hedges et al. (2010), which allows for the evaluation of estimates that are conceptually similar. Third, if longitudinal studies assess the effects of an eligible campaign on multiple time points rather than in pre/post, we will use (or compute) the effect size that would represent the change obtained between the baseline measurement and the first post‐measurement, making them comparable with other pre/post studies included in this review. Fourth, when studies report more than one effect size for an outcome associated with a specific subgroup (e.g., minors), we will calculate a weighted average effect for that subgroup. Fifth, when studies report data from interventions of multiple arms, data from all arms will be considered. Finally, if multiple publications report the same results derived from the same data set (e.g., an organization report later published in a peer‐reviewed journal), the most up‐to‐date study (usually that published in a peer‐reviewed journal) will be retained for the meta‐analysis.

#### 3.3.3 Selection of Studies

##### 3.3.3.1 Overview

To determine whether a study is eligible for inclusion in the review, we will use a screening process common to systematic reviews. We will first perform initial title and abstract screening, followed by a review of the full text of identified research.

##### 3.3.3.2 Title and Abstract Screening

As a first step in determining a study's eligibility for inclusion in the review, we will screen the titles and abstracts of all records we identify through our search practices described in Section 3.2. Specifically, following our removal of duplicate studies and obviously ineligible documents (e.g., book reviews), we will evaluate all titles and abstracts to determine whether they are eligible for inclusion in the review. See Table 7 for the coding form to be used to screen studies.

**Table 7:** Checklist for title and abstract screening.

Inclusion eligibility checklist	
1. There is a communicative intervention. This includes (but is not limited to): counter‐narratives, attitudinal inoculation, alternative narratives, education campaigns, suspicious activity reporting campaigns, etc.	Yes /No /Maybe
2. The intervention is developed/funded/implemented by a government organization or organization acting on behalf of a government.	Yes /No /Maybe
3. The intervention aims, among other things, to reduce engagement in violent extremism (as measured by primary or secondary outcomes).	Yes /No /Maybe
4. The study evaluates the intervention using an experimental or quasi‐experimental quantitative design, for example, an RCT control trial, a factorial design, or a pre‐/post‐ or longitudinal study.	Yes /No /Maybe
If all “yes” or “maybe”:	Proceed to full‐text screening (quantitatively eligible)
If a “no” is present:	
Study is a relevant review or background literature article.	Keep as a background report
Study contains a qualitative evaluation or an expert‐opinion evaluation of an eligible campaign.	Keep for the discussion
Else:	Discard (not eligible or useful)

Interrater agreement statistics will be computed using Fleiss (1971) kappa between coders for the initial 10% of search results, and if the interrater agreement is deemed unsatisfying, additional training will be provided to research assistants (or our inclusion/exclusion criteria will be clarified). Fleiss's kappa will then be computed again post‐training, and if still unsatisfying, another round of training/criteria review will be conducted. This process will be repeated iteratively until a satisfying kappa is obtained. Kappas for each stage will be presented in the final report.

##### 3.3.3.3 Full‐Text Eligibility Screening

We will screen the full text of documents deemed potentially eligible for inclusion in the review after screening their titles and abstracts. Our reading of the documents will allow us to remove studies from the review if they are obviously ineligible or are duplications of already included studies. We will also exclude studies that do not assess the effect of a government‐led, communication‐based intervention on salient outcomes. If a study is excluded at the full‐text reading step, its coding sheet will be only partially filled, with coders nevertheless required to answer the following: (a) Based on the full‐text reading, is the paper eligible for the systematic review? (b) If not, why? Whenever full‐text reading reveals that a study does not meet the inclusion/exclusion criteria, we will document the specific reason(s) for its exclusion. However, studies will not be excluded based on risk of bias alone.

Once a preliminary list of candidate studies is identified via full‐text eligibility screening, two members of the research team will make final decisions on the studies to be included in the systematic review. This will involve the independent reading of the candidate studies. Each of the two members of the research team will independently decide whether a study should be included in the final review. Studies that are agreed upon by both members of the team as appropriate will be included in the review. Studies that neither member of the team identifies as appropriate will not be included. When there are disagreements on inclusion, those disagreements will be resolved with discussion among the entire research team (not just the two team members who reviewed the preliminary list of candidate studies) such that the two initial reviewers will explain their decision to the rest of the research team. The remaining members of the research team will then vote on the study's inclusion. If disagreements remain at this stage, the main investigator will make the final decision.

The process by which studies were eliminated from contention for inclusion will be documented in a PRISMA flow chart, with explanations for exclusion included at each step.

#### 3.3.4 Data Extraction and Management

Documents that are selected for inclusion in the review following screening will be coded along several lines. Each retained study will be processed in an Excel coding sheet where the following information will be extracted:

- Document ID, title, authors, year of publication, and place published;
- Source of funding and declaration of conflicts of interests;
- Final eligibility (taking into consideration inclusion/exclusion criteria and risk of bias);
- Intervention/campaign characteristics:
  ◦ Name;
  ◦ Objectives;
  ◦ Country;
  ◦ Source;
  ◦ Ideologies targeted;
  ◦ Target audience;
  ◦ Platform/form of communication;
  ◦ Content (modules/activities);
  ◦ Reasons for implementation;
  ◦ Length and frequency of exposure; and
  ◦ Other relevant information.

- Study design (RCT, factorial design, pre/post, etc.);
- Data source (campaign participants, stakeholders, “big data”);
- Sample characteristics:
  ◦ Sample constitution procedure (survey, clinical observation, big data collection, etc.);
  ◦ Number of participants;
  ◦ Male/female/non‐binary split;
  ◦ Age;
  ◦ Country where data was collected;
  ◦ Ethno‐racial group;
  ◦ Education level;
  ◦ Employment status;
  ◦ Religious affiliation;
  ◦ Ideological affiliation (in relation to violent extremist ideas);
  ◦ Type of big data (tweets, likes, etc.); and
  ◦ Other relevant information.

- Measures:
  ◦ Independent variables (modules, interventions, etc.);
  ◦ Moderators (age, gender, ideology, etc.); and
  ◦ Dependent variables (primary/secondary outcomes).

- Quantitative results:
  ◦ Positive outcomes (effect sizes); and
  ◦ Negative outcomes (effect sizes).

- Overall, were the objectives of the campaign met?
- Recommendations of authors:
  ◦ Concerning the intervention/campaign;
  ◦ For practitioners/stakeholders;
  ◦ For future research;
  ◦ For policy; and
  ◦ Other recommendations.

- Limitations mentioned by authors;
- Limitations not mentioned by authors and found by the coder; and
- Coder ID and coding date.

In some cases, studies will report both quantitative and qualitative data. When this occurs, we will extract quantitative effect sizes and take note of qualitative results for our discussion.

#### 3.3.5 Assessment of Risk of Bias

Because of the state of the literature and ethical concerns in the PVE space as it relates to campaign evaluation, we anticipate that very few (or no) random allocation studies will be available. Furthermore, using tools made available by Cochrane, such as the Risk of Bias 2 (Higgins et al. 2019), would likely not be fit for purpose as they are from the medical field. Therefore, to accommodate the multiplicity of designs present in the PVE field, we will use the general and quantitative sections of the Mixed Methods Appraisal Tool (MMAT; Hong et al. 2018) to assess the reliability of results contained in eligible quantitative papers. The MMAT has already been used in a Campbell Collaboration review in the PVE space with good results (Madriaza et al. 2024).

Using this tool, we will rate studies as being characterized as having a high, low, or unclear risk of bias. Specifically, two members of the review team will perform the assessment of potential bias to independently confirm (or refute) the presence of bias in the collected data. Studies with a high risk of bias will be excluded at the coding sheet level, and studies with an unclear risk of bias will be discussed among study authors until a verdict is reached (include or exclude).

##### 3.3.5.1 Assessment of Publication or Small‐Study Biases

We aim to mitigate publication bias through the comprehensive inclusion of gray literature. To further minimize the potential impact of publication or small‐study biases, however, we will employ the “trim and fill” method (Duval and Tweedie 2000a, 2000b) and the Egger regression test (Egger et al. 1997). The trim and fill method allows us to identify asymmetric funnel plots as potential markers of publication bias. As a first step, we will trim the smallest studies from the side of the funnel responsible for the asymmetry. Next, we will impute effect sizes on the opposite side of the funnel using a predetermined estimator. Finally, we will revise the overall effect size by adding the imputed studies, repeating the procedure until the funnel plot achieves symmetry around the new effect size (Borenstein et al. 2009). In the Egger regression analysis, we will treat a significant intercept estimate greater than 0 as a marker of potential publication bias. Nevertheless, to ensure reliable results, both methods will need an adequate number of studies in each meta‐analysis.

#### 3.3.6 Data Analysis

The data to be synthesized will be the effects of government‐led, communication‐based interventions on salient outcomes, such as violent radical behaviors, intentions, attitudes, or beliefs. Effect sizes will be synthesized with meta‐analytical techniques when conceptually appropriate. Effect sizes of interventions will be meta‐analyzed and graphically represented in forest plots when:

- They affect the same types of outcomes (see Table 1);
- They are assessed in the same type of experimental design (RCT, factorial design, pre/post, longitudinal); and
- They result from the same type of intervention (e.g., counter‐narrative campaign, attitudinal inoculation campaign, education campaigns, suspicious activity reporting, etc.).

Moderator variables related to the type of messaging platform (e.g., social media, traditional media, Web 1.0 sites), targeted audience, targeted ideology (e.g., far right, religiously inspired, far left), or setting will be taken into consideration in moderation analyses (see Assessment and investigation of heterogeneity below).

##### 3.3.6.1 Effect Sizes Amenable to Synthesis

For studies that report quantitative data concerning the effect of a government‐led, communication‐based intervention on salient outcomes, we will extract those data and incorporate them into our meta‐analysis. In meta‐analysis, it is necessary to convert available data to a common statistical metric. For the current review, we will first convert all estimates of association to Cohen's *d*. This systematic review will follow the same analytical strategy as that of Madriaza et al. (2024), conducted under Campbell guidance.

For experimental studies (randomized and not randomized) using two independent groups, we will estimate the standardized mean difference as follows:

$$d=\frac{\bar{X_{1}}-\bar{X_{2}}}{S_{\mathrm{pooled}}},$$

where *X*_1_ is the mean of the treatment sample, *X*_2_ is the mean of the control group, and S_pooled_ is the standard deviation within groups, pooled across groups and defined by:

$$S_{\mathrm{pooled}}=\sqrt{\frac{\left(n_{1}-1\right)S_{1}^{2}+\left(n_{2}-1\right)S_{2}^{2}}{n_{1}+n_{2}-2}},$$

where *n*_1_ and *n*_2_ are the sample sizes in the two groups, and *S*_1_ and *S*_2_ are the standard deviations in the two groups (Borenstein et al. 2009).

For studies that report effect sizes as correlation coefficients, we will then convert the correlation coefficient *r* to Cohen's *d* using this formula:

$$d=\frac{2r}{\sqrt{1-r^{2}}}.$$

For studies in which we only have access to the coefficients of multivariate statistical models, partial effect sizes will be estimated using the Online calculator, following the formulas below inspired by Lipsey and Wilson (2001):

- In cases where the independent and dependent variables are dichotomous variables and only *β* is reported, Cohen's *d* will be calculated as follows:

$$d=\beta (\frac{\sqrt{3}}{\pi }).$$

- For linear regression models where both independent and dependent variables are continuous, *r* will be first calculated and then converted to *d* according to the previous formula. In this situation, *r* will be calculated as follows:

$$r=\frac{SD_{X}\beta }{{\mathrm{SD}}_{Y}}.$$

- In cases in which the standard deviation is not reported, particularly in situations where the independent variables are dichotomous, and the dependent variables are continuous, as well as in situations in which the independent variables are ordinal or continuous, and the dependent variables are dichotomous, we will proceed to calculate *r* using the ratio *t* = *β*/SE and the following formula:

$$r=t/\sqrt{t^{2}+n-2}.$$

##### 3.3.6.2 Statistical Procedures

Statistical analysis will, again, be based on the strategy put together by Madriaza et al. (2022). Meta‐analysis will be performed using Biostat Comprehensive Meta‐Analysis (CMA) version 4 software (Borenstein et al. 2009). Because the data from the studies included in this systematic review will be from samples from different populations, random‐effects models will be used to account for this heterogeneity.

As mentioned, we will organize effect sizes according to the type of outcome, type of experimental design, and type of intervention. We will present the results of meta‐analyses as standardized difference‐in‐means with 95% confidence intervals in a series of ordered tables. Furthermore, we will use Robust Variance Estimation (RVE) to handle dependent effect sizes in a meta‐analysis when studies report multiple conceptually related effects. RVE will be implemented using the method described by Hedges et al. (2010), through the *robumeta* package in R. This method will adjust the standard errors of the global average effect estimators to account for the dependency between multiple effects within studies.

##### 3.3.6.3 Sensitivity Analysis

We will apply the “one study removed” technique provided by the CMA software to detect and examine possible outliers in each meta‐analysis. This technique is only reliable and informative when the meta‐analyses have at least three studies. Therefore, we will only report the results of meta‐analyses that meet this criterion. We will inspect the results to identify whether a single effect size exerts a large influence on heterogeneity. To do so, we will examine whether the elimination of a single study results in a nonsignificant *Q* value.

##### 3.3.6.4 Assessment and Investigation of Heterogeneity

We will use several methods to evaluate the heterogeneity of the data in each meta‐analysis. These will include Cochran's *Q* (and its corresponding *χ* ^2^ value), *τ* ^2^, the *I* ^2^ statistic, and the prediction interval. A significant *Q* value suggests possible heterogeneity. On the other hand, an *I* ^2^ value of zero indicates no heterogeneity. Following Borenstein (2019), we will use the prediction interval to determine the degree of data dispersion within a meta‐analysis.

When we find significant heterogeneity and there are enough studies, we will try to identify its potential sources by conducting meta‐regressions for continuous variables and moderation analyses for categorical variables. We will only perform these analyses when meta‐analyses have at least 5 studies. We plan to investigate heterogeneity for the following control variables: type of messaging platform, targeted audience, targeted ideology, and setting.

##### 3.3.6.5 Length of Intervention Potential Issues

Given the nature of government‐led, communication‐based interventions, the most likely issue we will encounter related to units of analysis will concern the length of time between exposure to the intervention and measurement of salient outcomes, as well as the possibility that some interventions will be evaluated at multiple posttest time points while others will be measured at a single posttest time point. In cases where interventions are evaluated at multiple points in time, we will prioritize the effect sizes that represent the change between the baseline measurement and the first post‐measurement, thus replicating pre/post designs.

##### 3.3.6.6 Dealing With Missing Data

All studies included in the meta‐analysis will need to report sufficient data to compute an effect size reflecting the relationship between the intervention and an eligible outcome. In the absence of directly reported effect sizes, we will use sample sizes, *t*‐ or *F*‐test scores, and associated *p*‐values to compute effect sizes ourselves.

If we are unable to obtain or compute the missing data, we will nonetheless retain the study for description in the final report. These studies cannot be used to make deductions about intervention effectiveness, but their inclusion will allow us to provide a more comprehensive account of the government‐led communication‐based intervention landscape.

## Author Contributions

Completion of the review will be broken down into three elements:

- First, Wynnpaul Varela, Sébastien Brouillette‐Alarie, and Kurt Braddock—with assistance from Campbell Editor Liz Eggins—will supervise the retrieval of documents, selection of studies, and extraction of data from those studies, along with two research assistants.
- Second, statistical analyses will be performed by authors Sébastien Brouillette‐Alarie and Pablo Madriaza.
- Finally, the content of the final report will be compiled and written by Ghayda Hassan, Sébastien Brouillette‐Alarie, Kurt Braddock, Sarah Carthy, Paul Gill, Pablo Madriaza, and Wynnpaul Varela.

## Conflicts of Interest

Kurt Braddock and Sarah Carthy have published research that is closely linked with the review topic. As a result, studies they have performed may appear in the systematic review. The other authors declare no conflicts of interest.

## Preliminary Timeframe

We plan to submit the final review to Campbell Corporation in December 2025.

## Plans for Updating the Review

Ghayda Hassan, Wynnpaul Varela, Sébastien Brouillette‐Alarie, and Kurt Braddock will be responsible for updating this review if needed, which is anticipated to occur every 3–5 years.

## Sources of Support

### Internal Sources

Funding for this review is provided by a Campbell Collaboration grant awarded to the authors.

### External Sources

There is no external funding associated with the performance of this systematic review.

## Supporting information

Supporting information.
