# Supplementary material for: PROTOCOL: Government‐Led Communication Campaigns for Reducing Violent Extremism − A Systematic Review
Source: Campbell Syst Rev. 2025 Apr 1;21(2):e70031. doi: 10.1002/cl2.70031 (PMC11959302; doi:10.1002/cl2.70031)
Supplement: Supplementary file 1 — Supporting information. [file CL2-21-e70031-s001.docx]

**Appendix A**

**EBSCOhost Databases Search**

Academic Search Complete, Communication and Mass Media Complete, Communication Abstracts, Criminal Justice Abstracts, Military and Government Collection, PsycARTICLES, PsycEXTRA, Psychology and Behavioral Sciences Collection, PsycINFO

Trial performed on Academic Search Complete, February 23, 2025

S1: TI ( (messag* OR communic* OR fram* OR narrative* OR argument*) W3 (initiative* OR program* OR project* OR campaign* OR interv* OR counter* OR contest* OR alter* OR anti OR inoculat* OR persua* OR dissua* OR awareness OR inform* OR educat* OR prevent*) ) OR AB ( (messag* OR communic* OR fram* OR narrative* OR argument*) W3 (initiative* OR program* OR project* OR campaign* OR interv* OR counter* OR contest* OR alter* OR anti OR inoculat* OR persua* OR dissua* OR awareness OR inform* OR educat* OR prevent*) ) OR SU ( (messag* OR communic* OR fram* OR narrative* OR argument*) W3 (initiative* OR program* OR project* OR campaign* OR interv* OR counter* OR contest* OR alter* OR anti OR inoculat* OR persua* OR dissua* OR awareness OR inform* OR educat* OR prevent*) ) 89,884

S2: TI ( (initiative* OR program* OR project* OR campaign* OR interv*) W3 (counter* OR contest* OR alter* OR anti OR inoculat* OR persua* OR dissua* OR awareness OR inform* OR educat* OR prevent*) ) OR AB ( (initiative* OR program* OR project* OR campaign* OR interv*) W3 (counter* OR contest* OR alter* OR anti OR inoculat* OR persua* OR dissua* OR awareness OR inform* OR educat* OR prevent*) ) OR SU ( (initiative* OR program* OR project* OR campaign* OR interv*) W3 (counter* OR contest* OR alter* OR anti OR inoculat* OR persua* OR dissua* OR awareness OR inform* OR educat* OR prevent*) ) 154,310

S3: TI campaign* OR AB campaign* OR SU campaign* 306,381

S4: S1 OR S2 OR S3  533,321

S5: TI ( (council* OR government* OR minist* OR state* OR provinc* OR nation* OR department* OR federal*) ) OR AB ( (council* OR government* OR minist* OR state* OR provinc* OR nation* OR department* OR federal*) ) OR SU ( (council* OR government* OR minist* OR state* OR provinc* OR nation* OR department* OR federal*) ) 8,833,862

S6: S4 AND S5 249,694

S7: TI ( (radicali* OR “alt left” OR “alt-left” OR “alt right” OR “alt-right” OR anarch* OR anticapitalis* OR “anti-capitalis* OR “anti capitalis*” OR antifas* OR “anti fas*” OR “anti-fas*” OR “anti islam*” OR “anti-islam* OR “anti muslim*” OR “anti-muslim*” OR antisemiti* OR “anti-semiti* OR “anti semiti*” OR blackpill OR “black-pill” OR “black pill” OR ecoterror* OR “eco-terror*” OR “eco terror*” OR ecoviolen* OR “eco-violen*” OR “eco violen*” OR “environmental* violen*” OR “extreme right” OR extremis* OR fanatici* OR “far left” OR “far-left” OR “far right” OR “far-right” OR fascis* OR “foreign fight*” OR fundamentalis* OR “hate crime*” OR “ideological* violen*” OR incel OR indoctrinat* OR insurgen* OR islamis* OR islamophob* OR jihadis* OR “left wing” OR “left-wing” OR “male supremac*” OR “mass shoot*” OR misogyn* OR neonazi* OR “neo nazi*” OR redpill OR “red-pill” OR “red pill” OR “right wing” OR “right-wing” OR salafi* OR “school shoot*” OR “scientific racis*” OR supremis* OR terroris* OR “white supremac*” OR zionis*) ) OR AB ( (radicali* OR “alt left” OR “alt-left” OR “alt right” OR “alt-right” OR anarch* OR anticapitalis* OR “anti-capitalis* OR “anti capitalis*” OR antifas* OR “anti fas*” OR “anti-fas*” OR “anti islam*” OR “anti-islam* OR “anti muslim*” OR “anti-muslim*” OR antisemiti* OR “anti-semiti* OR “anti semiti*” OR blackpill OR “black-pill” OR “black pill” OR ecoterror* OR “eco-terror*” OR “eco terror*” OR ecoviolen* OR “eco-violen*” OR “eco violen*” OR “environmental* violen*” OR “extreme right” OR extremis* OR fanatici* OR “far left” OR “far-left” OR “far right” OR “far-right” OR fascis* OR “foreign fight*” OR fundamentalis* OR “hate crime*” OR “ideological* violen*” OR incel OR indoctrinat* OR insurgen* OR islamis* OR islamophob* OR jihadis* OR “left wing” OR “left-wing” OR “male supremac*” OR “mass shoot*” OR misogyn* OR neonazi* OR “neo nazi*” OR redpill OR “red-pill” OR “red pill” OR “right wing” OR “right-wing” OR salafi* OR “school shoot*” OR “scientific racis*” OR supremis* OR terroris* OR “white supremac*” OR zionis*) ) OR SU ( (radicali* OR “alt left” OR “alt-left” OR “alt right” OR “alt-right” OR anarch* OR anticapitalis* OR “anti-capitalis* OR “anti capitalis*” OR antifas* OR “anti fas*” OR “anti-fas*” OR “anti islam*” OR “anti-islam* OR “anti muslim*” OR “anti-muslim*” OR antisemiti* OR “anti-semiti* OR “anti semiti*” OR blackpill OR “black-pill” OR “black pill” OR ecoterror* OR “eco-terror*” OR “eco terror*” OR ecoviolen* OR “eco-violen*” OR “eco violen*” OR “environmental* violen*” OR “extreme right” OR extremis* OR fanatici* OR “far left” OR “far-left” OR “far right” OR “far-right” OR fascis* OR “foreign fight*” OR fundamentalis* OR “hate crime*” OR “ideological* violen*” OR incel OR indoctrinat* OR insurgen* OR islamis* OR islamophob* OR jihadis* OR “left wing” OR “left-wing” OR “male supremac*” OR “mass shoot*” OR misogyn* OR neonazi* OR “neo nazi*” OR redpill OR “red-pill” OR “red pill” OR “right wing” OR “right-wing” OR salafi* OR “school shoot*” OR “scientific racis*” OR supremis* OR terroris* OR “white supremac*” OR zionis*) ) 41,219

S8: TI ( (lone) W2 (actor* OR offend* OR wolf*) ) OR AB ( (lone) W2 (actor* OR offend* OR wolf*) ) OR SU ( (lone) W2 (actor* OR offend* OR wolf*) ) 572

S9: TI ( (violen*) W2 (political* OR racis* OR religio* OR separatis*) ) OR AB ( (violen*) W2 (political* OR racis* OR religio* OR separatis*) ) OR SU ( (violen*) W2 (political* OR racis* OR religio* OR separatis*) ) 2,805

S10: TI ( (radical*) W2 (group* OR ideolog* OR left* OR movement* OR right*) ) OR AB ( (radical*) W2 (group* OR ideolog* OR left* OR movement* OR right*) ) OR SU ( (radical*) W2 (group* OR ideolog* OR left* OR movement* OR right*) ) 6,179

S11: TI ( (suicid*) W2 (attack* OR bomb*) ) OR AB ( (suicid*) W2 (attack* OR bomb*) ) OR SU ( (suicid*) W2 (attack* OR bomb*) ) 7,305

S12: TI ( (violen*) W2 (racis* OR separatis*) ) OR AB ( (violen*) W2 (racis* OR separatis*) ) OR SU ( (violen*) W2 (racis* OR separatis*) ) 475

S13: S7 OR S8 OR S9 OR S10 OR S11 OR S12 56,789

S14: S6 AND S13 1,275

S15: TI ( (evaluat* OR assess* OR apprais* OR effectiv* OR efficac* OR investigat* OR impact* OR experiment* OR trial* OR quasiexperiment* OR “quasi-experiment*” OR “quasi experiment*” OR random* OR RCT OR analy* OR measur* OR outcome*) ) OR AB ( (evaluat* OR assess* OR apprais* OR effectiv* OR efficac* OR investigat* OR impact* OR experiment* OR trial* OR quasiexperiment* OR “quasi-experiment*” OR “quasi experiment*” OR random* OR RCT OR analy* OR measur* OR outcome*) ) OR SU ( (evaluat* OR assess* OR apprais* OR effectiv* OR efficac* OR investigat* OR impact* OR experiment* OR trial* OR quasiexperiment* OR “quasi-experiment*” OR “quasi experiment*” OR random* OR RCT OR analy* OR measur* OR outcome*) ) 20,048,597

S16: S14 AND S15  474

Limiters - Publication Date: -20241231

**RESULTS: 474**

**Appendix B**

**ProQuest Databases Search**

Applied Social Sciences Index and Abstracts (ASSIA), Criminal Justice Abstracts, Dissertations and Theses Index, International Bibliography of the Social Sciences (IBSS), National Criminal Justice Reference Service (NCJRS), Policy File Index, ProQuest Criminal Justice, ProQuest Political Science, ProQuest Social Science, ProQuest Sociology, Social Services Abstracts, Sociological Abstracts, Worldwide Political Science Abstracts

Trial performed on Sociological Abstracts, February 23, 2025

S1: noft((messag* OR communic* OR fram* OR narrative* OR argument*) W/3 (initiative* OR program* OR project* OR campaign* OR interv* OR counter* OR contest* OR alter* OR anti OR inoculat* OR persua* OR dissua* OR awareness OR inform* OR educat* OR prevent*)) OR noft((initiative* OR program* OR project* OR campaign* OR interv*) W/3 (counter* OR contest* OR alter* OR anti OR inoculat* OR persua* OR dissua* OR awareness OR inform* OR educat* OR prevent*)) OR noft(campaign*) 22,265

S2: noft((council* OR government* OR minist* OR state* OR provinc* OR nation* OR department* OR federal*)) **AND** noft((evaluat* OR assess* OR apprais* OR effectiv* OR efficac* OR investigat* OR impact* OR experiment* OR trial* OR quasiexperiment* OR "quasi-experiment*" OR "quasi experiment*" OR random* OR RCT OR analy* OR measur* OR outcome*)) 479,829

S3: noft((radicali* OR "alt left" OR "alt-left" OR "alt right" OR "alt-right" OR anarch* OR anticapitalis* OR "anti-capitalis*" OR "anti capitalis*" OR antifas* OR "anti fas*" OR "anti-fas*" OR "anti islam*" OR "anti-islam*" OR "anti muslim*" OR "anti-muslim*" OR antisemiti* OR "anti-semiti*" OR "anti semiti*" OR blackpill OR "black-pill" OR "black pill" OR ecoterror* OR "eco-terror*" OR "eco terror*" OR ecoviolen* OR "eco-violen*" OR "eco violen*" OR "environmental* violen*" OR "extreme right" OR extremis* OR fanatici* OR "far left" OR "far-left" OR "far right" OR "far-right" OR fascis* OR "foreign fight*" OR fundamentalis* OR "hate crime*" OR "ideological* violen*" OR incel OR indoctrinat* OR insurgen* OR islamis* OR islamophob* OR jihadis* OR "left wing" OR "left-wing" OR "male supremac*" OR "mass shoot*" OR misogyn* OR neonazi* OR "neo nazi*" OR redpill OR "red-pill" OR "red pill" OR "right wing" OR "right-wing" OR salafi* OR "school shoot*" OR "scientific racis*" OR supremis* OR terroris* OR "white supremac*" OR zionis*)) 66,300

S4: noft((lone) W/2 (actor* OR offend* OR wolf*)) OR noft((violen*) W/2 (political* OR racis* OR religio* OR separatis*)) OR noft((radical*) W/2 (group* OR ideolog* OR left* OR movement* OR right*)) OR noft((suicid*) W/2 (attack* OR bomb*)) OR noft((violen*) W/2 (racis* OR separatis*)) 110

S5: [S3] OR [S4] 66,312

S6: [S1] AND [S2] AND [S5] 936

Limited by: Date: Before 31 December 2024

**RESULTS: 936 (914 after 22 magazines excluded)**

**Appendix C**

**Web of Science Database Search**

Conference Proceedings Index: Social Sciences and Humanities, Emerging Sources Citation Index, Social Sciences Citation Index

Trial performed on all the above databases on February 23, 2025

S1: (messag* OR communic* OR fram* OR narrative* OR argument*) NEAR/3 (initiative* OR program* OR project* OR campaign* OR interv* OR counter* OR contest* OR alter* OR anti OR inoculat* OR persua* OR dissua* OR awareness OR inform* OR educat* OR prevent*) (Topic) or (initiative* OR program* OR project* OR campaign* OR interv*) NEAR/3 (counter* OR contest* OR alter* OR anti OR inoculat* OR persua* OR dissua* OR awareness OR inform* OR educat* OR prevent*) (Topic) or campaign* (Topic) and 1900-2024 (Year Published) 1,014,656

S2: (radicali* OR "alt left" OR "alt-left" OR "alt right" OR "alt-right" OR anarch* OR anticapitalis* OR "anti-capitalis*" OR "anti capitalis*" OR antifas* OR "anti fas*" OR "anti-fas*" OR "anti islam*" OR "anti-islam*" OR "anti muslim*" OR "anti-muslim*" OR antisemiti* OR "anti-semiti*" OR "anti semiti*" OR blackpill OR "black-pill" OR "black pill" OR ecoterror* OR "eco-terror*" OR "eco terror*" OR ecoviolen* OR "eco-violen*" OR "eco violen*" OR "environmental* violen*" OR "extreme right" OR extremis* OR fanatici* OR "far left" OR "far-left" OR "far right" OR "far-right" OR fascis* OR "foreign fight*" OR fundamentalis* OR "hate crime*" OR "ideological* violen*" OR incel OR indoctrinat* OR insurgen* OR islamis* OR islamophob* OR jihadis* OR "left wing" OR "left-wing" OR "male supremac*" OR "mass shoot*" OR misogyn* OR neonazi* OR "neo nazi*" OR redpill OR "red-pill" OR "red pill" OR "right wing" OR "right-wing" OR salafi* OR "school shoot*" OR "scientific racis*" OR supremis* OR terroris* OR "white supremac*" OR zionis*) (Topic) or (lone) NEAR/2 (actor* OR offend* OR wolf*) (Topic) or (violen*) NEAR/2 (political* OR racis* OR religio* OR separatis*) (Topic) or (radical*) NEAR/2 (group* OR ideolog* OR left* OR movement* OR right*) (Topic) or (suicid*) NEAR/2 (attack* OR bomb*) (Topic) or (violen*) NEAR/2 (racis* OR separatis*) (Topic) and 1900-2024 (Year Published) 191,042

S3: (council* OR government* OR minist* OR state* OR provinc* OR nation* OR department* OR federal*) (Topic) and (evaluat* OR assess* OR apprais* OR effectiv* OR efficac* OR investigat* OR impact* OR experiment* OR trial* OR quasiexperiment* OR "quasi-experiment*" OR "quasi experiment*" OR random* OR RCT OR analy* OR measur* OR outcome*) (Topic) and 1900-2024 (Year Published) 6,618,261

S4: #1 AND #2 AND #3

**RESULTS: 3,226**

**Appendix D**

**PsycINFO OVID (Database) Search**

Trial performed on February 28, 2025

1 (((messag* or communic* or fram* or narrative* or argument*) adj3 (initiative* or program* or project* or campaign* or interv* or counter* or contest* or alter* or anti or inoculat* or persua* or dissua* or awareness or inform* or educat* or prevent*)) or ((initiative* or program* or project* or campaign* or interv*) adj3 (counter* or contest* or alter* or anti or inoculat* or persua* or dissua* or awareness or inform* or educat* or prevent*)) or campaign*).ab,hw,id,mh,ot,ti. 287283

2 (council* or government* or minist* or state* or provinc* or nation* or department* or federal*).ab,hw,id,mh,ot,ti. 993646

3 (radicali* or "alt left" or "alt-left" or "alt right" or "alt-right" or anarch* or anticapitalis* or "anti-capitalis*" or "anti capitalis*" or antifas* or "anti fas*" or "anti-fas*" or "anti islam*" or "anti-islam*" or "anti muslim*" or "anti-muslim*" or antisemiti* or "anti-semiti*" or "anti semiti*" or blackpill or "black-pill" or "black pill" or ecoterror* or "eco-terror*" or "eco terror*" or ecoviolen* or "eco-violen*" or "eco violen*" or "environmental* violen*" or "extreme right" or extremis* or fanatici* or "far left" or "far-left" or "far right" or "far-right" or fascis* or "foreign fight*" or fundamentalis* or "hate crime*" or "ideological* violen*" or incel or indoctrinat* or insurgen* or islamis* or islamophob* or jihadis* or "left wing" or "left-wing" or "male supremac*" or "mass shoot*" or misogyn* or neonazi* or "neo nazi*" or redpill or "red-pill" or "red pill" or "right wing" or "right-wing" or salafi* or "school shoot*" or "scientific racis*" or supremis* or terroris* or "white supremac*" or zionis* or (lone adj2 (actor* or offend* or wolf*)) or (violen* adj2 (political* or racis* or religio* or separatis*)) or (radical* adj2 (group* or ideolog* or left* or movement* or right*)) or (suicid* adj2 (attack* or bomb*)) or (violen* adj2 (racis* or separatis*))).ab,hw,id,mh,ot,ti. 33409

4 (evaluat* or assess* or apprais* or effectiv* or efficac* or investigat* or impact* or experiment* or trial* or quasiexperiment* or "quasi-experiment*" or "quasi experiment*" or random* or RCT or analy* or measur* or outcome*).ab,hw,id,mh,ot,ti. 3992544

5 1 and 2 and 3 and 4 790

Limited by: Date: Before 31 December 2024

**RESULTS: 790**
